# Expression of progranulin (GP88) protein appears as an independent prognostic factor for clinical progression in high-risk prostate cancer patients

**DOI:** 10.1038/s41598-026-52197-0

**Published:** 2026-06-11

**Authors:** Renata Dubrovska, Markus Eckstein, Rudolf Jung, Charis Kalogirou, Burkhard Kneitz, Martin Spahn, Marianna Kruithof-de Julio, Ginette Serrero, Binbin Yue, Carol Geppert, Robert Stöhr, Arndt Hartmann, Bernd Wullich, Verena Lieb, Helge Taubert, Sven Wach

**Affiliations:** 1 Department of Urology and Pediatric Urology, Universitätsklinikum Erlangen, Friedrich-Alexander-Universität Erlangen-Nürnberg, 91054 Erlangen, Germany; 2 Department of Pathology, Universitätsklinikum Erlangen, Friedrich-Alexander-Universität Erlangen-Nürnberg, 91054 Erlangen, Germany; 3 Comprehensive Cancer Center Erlangen-EMN (CCC ER-EMN), 91054 Erlangen, Germany; 4 Department of Urology and Paediatric Urology, University Hospital Würzburg, 97080 Würzburg, Germany; 5 Lindenhofspital, Bern, Switzerland; 6 Department of Urology, Inselspital, Bern University Hospital, Bern, Switzerland; 7 A&G Pharmaceutical Inc., Columbia, MD 21045 USA; 8 Program in Oncology, University of Maryland Greenebaum Comprehensive Cancer Center, Baltimore, MD 21201 USA

**Keywords:** Prostate cancer, Progranulin, GP88, Immunohistochemistry, Protein expression, Clinical progression, Clinical progression free survival, Biomarkers, Cancer, Oncology

## Abstract

Prostate cancer (PCa), the second most common cancer, is still a major cause of morbidity and mortality among men worldwide. A particularly aggressive group of PCa, where prognostic biomarkers are still needed, are clinically defined high-risk PCa. We had previously shown that the survival and proliferation factor progranulin (GP88) is a prognostic marker for primary PCa. We aimed in this study to characterize GP88 protein expression in high-risk PCa by immunohistochemistry and to examine its association with prognosis. Immunohistochemical staining for GP88 was performed with TMA of samples from 94 high-risk PCa patients using an H-score. GP88 staining was in a range of 3.69 to 252.51 (median: 109.28). The association of GP88 staining with prognosis was examined by survival analyses (Kaplan–Meier, multivariate Cox’s regression analysis). Elevated GP88 expression was associated with a shorter clinical progression-free survival (CPFS) in all PCa patients (*P* = 0.043), and in the following patient subgroups: Elder PCa patients (> 67 years; *P* = 0.020), patients with pT3 tumors (*P* = 0.008), with Gleason scores GS5 and GS6 (*P* = 0.033 and *P* = 0.030), and patients without radiation therapy (*P* = 0.009) at considering that only four patients received an adjuvant radiation therapy. In a multivariate Cox’s regression analysis, increased GP88 protein expression (RR = 2.98; *P* = 0.049) and the preoperative PSA level (RR = 5.29; *P* = 0.030) appeared as independent prognostic factors for clinical progression. Interestingly, lower GP88 staining in corresponding low Gleason lesions was correlated with the presence of immune cells, suggesting an immune cell suppressive effect of GP88. Altogether, Progranulin (GP88) protein positivity appears very likely to be an independent prognostic factor for clinical progression in high-risk PCa patients.

**Supplementary Information:**

The online version contains supplementary material available at 10.1038/s41598-026-52197-0.

## Introduction

Prostate cancer (PCa) is the second most common cancer in men worldwide with 1.46 million new cases and 375,000 deaths in 2020^1^. PCa in its early localized stages is usually treated with curative intent by local therapy such as radical prostatectomy, external beam radiation therapy or brachytherapy. But eventually, progression towards a hormone refractory or castration resistant stage (CRPC) or its metastatic form (mCRPC) can occur, which is the major cause of morbidity and mortality. Biomarker-driven approaches for molecular profiling and therapy stratification of mCRPC have been described recently^2^. They identified distinct genomic alterations, e.g., defects in homologous recombination repair genes and mismatch repair alterations, which guide therapeutic decision making. In addition, novel treatment modalities are being explored, including androgen receptor (AR) degraders, antibody–drug conjugates, T-cell engagers, and epigenetic modulators^2^.

Of special interest for the development of mCRPC are high-risk PCa patients with at least one of the following risk factors: prostate-specific antigen (PSA) > 20 ng/ml, cT3, biopsy Gleason scores 8–10 since these patients have the highest risk to progress to mCRPC. High-risk PCa accounts for 50–75% of 10-year relapse after primary treatment^3^. A Risk-Stratification of High-Risk Prostate Cancer by clinical parameters has been reported^4–6^. Only a few biomarkers for the characterization of high-risk PCa have been reported, such as tenascin C (TNC), miR-205, miR-221, squalene epoxidase (SQL) or sterol-O-acyl transferase 1 (SOAT1)^7–9^. Altogether, there is still a need for the identification of molecular biomarkers that can better predict the clinical progression of high-risk PCa patients.

Progranulin (GRN, also known as GP88, PGRN, PCDGF, Acrogranin, Proepithelin) is an 88 kDa glycoprotein. It is a pleiotrophic growth factor that stimulates different cellular processes as angiogenesis, cell proliferation, migration, invasion, immune evasion, survival and anticancer drug resistance^10,11^. Progranulin will be referred as GP88 in the rest of the manuscript.

GP88 overexpression has been reported for many tumor entities as cancer of the breast, brain, ovary, uterus, esophagus, liver, lung, kidney, bladder, hematologic system and prostate as well as mostly as associated with poor prognosis of cancer patients^11^.

GP88 is involved in the activation and/or regulation of different cancer-associated pathways^12^, as the ERK1/2 pathway in mouse breast cancer^13^, and in humans in colorectal cancer^14,15^, gastric cancer^16^, and the PI3K/Akt/mTOR pathway in cervical cancer^17^ and ovarian cancer^18^. On the other hand, inhibition of these pathways can reduce GP88 levels, what offers a possibility for early therapy monitoring^18^.

In a previous report, Pan et al. showed only a weak GP88 protein expression in normal prostate tissues, increased expression as early as at the development of prostatic intraepithelial neoplasia (PIN) and significantly elevated expression in invasive PCa^19^. We have shown that GP88 expression in the serum of PCa patients is associated with poor overall survival^20^. In addition, detection of GP88 protein in PCa tissue by immunohistochemistry was associated with poor overall, recurrence free and cancer specific survival^21^.

In this study, we were interested to determine whether GP88 expression was associated with prognosis in defined high-risk PCa patients. We studied GP88 by immunohistochemistry in high-risk PCa patients from the European Multicenter Prostate Cancer Clinical and Translational Research Group (EMPaCT) TMA cohort. The EMPaCT TMA and research applications have been described previously in detail^4,7,8,22–26^. We could find that overexpression of GP88 was significantly associated with the clinical progression-free survival (CPFS) of high-risk PCa patients.

## Materials and methods

### Patients and material

Patient prostate tissues in this study originated from a part of the tissue micro array TMA2 described in detail in Kalogirou et al.^8^. This TMA is part of the European Multicenter Prostate Cancer Clinical and Translational Research Group (EMPaCT) cohort^25^. Briefly, TMA2 comprises PCa patients who have undergone radical prostatectomy between 1987 and 2005 at the University Hospital Würzburg, Germany and the University Hospital Leuven, Belgium, and were considered as high-risk disease (i.e., at least one of the following risk factors: prostate-specific antigen (PSA) > 20 ng/ml, cT3, biopsy Gleason score of 8–10) treated by radical prostatectomy (RP) and pelvic lymph node dissection (PLND). The assembly of TMA2 was approved by the respective local Ethics Committees (No. 59/04 and B322201214832) (Table 1).

### Immunohistochemistry and analysis

For the GP88 protein expression study, a manual immunohistochemistry (IHC) protocol was applied as previously described^27^. Briefly, after heat pretreatment at 120 °C for 5 min with TE buffer, pH 9, and peroxidase blocking (Dako; Hamburg, Germany), a primary antibody against GP88 (monoclonal mouse anti-GP88/PGRN antibody, Cat. No. AG10009; Precision Antibody, A&G Pharmaceutical, Columbia, MD, USA) was applied for 30 min. Detailed information about the specificity and validation of the GP88/PGRN antibody is provided in the Supplementary Material and Methods. Cytoplasmic GP88 expression was evaluated in a semi-quantitative way as H-score [scale:0–300] by two examiners (M.E., R.D.). Immune cells were quantified manually in Qupath^28^ by visual inspection of the image sections. Briefly, using digitized pathological slides, a standardized ellipse covering 1.07mm^2^ in the tumor area was defined for cell counting. We used the built-in method “Positive cell detection”. The evaluation was performed as a binary classification scheme (0 = no detectable immune cells; 1 = immune cells present) by two examiners (M.E., R.D.).

### Statistical analyses

The correlations between the H-score of GP88 staining and clinico-pathological data were calculated using Spearman’s bivariate correlation or the Chi^2^-test. Correlations between GP88 staining and the presence of immune cells was calculated by the Fisher’s exact test. To assess the association between the H-score of the GP88 staining and the clinical progression free survival (CPFS), a ROC analysis was performed. Afterwards, the Youden index was calculated to define optimal H-score cutoff values for separating CPFS. The associations of the expression of GP88 with CPFS was determined by univariate (Kaplan–Meier analysis and Cox’s regression hazard models) and multivariate analyses (Cox’s regression hazard models with backward variable selection, finally adjusted for preoperative PSA). A p-value < 0.05 was considered statistically significant. The statistical analyses were performed with the SPSS 29.0.1.0 software package (SPSS Inc., Chicago, IL, USA).

Multivariate Cox’s regression analysis was adjusted for clinico-pathological parameters, i.e., age at operation, pathological tumor stage (pT), pathological lymph node stage (pN), preoperative serum PSA, Gleason score of the surgical specimen, surgical margin status, adjuvant androgen deprivation therapy within 3 months after surgery, adjuvant radiation therapy of the prostatic bed within 3 months after surgery.

## Results

### GP88 expression and correlation with clinico-pathological parameters

**Fig. 1:**
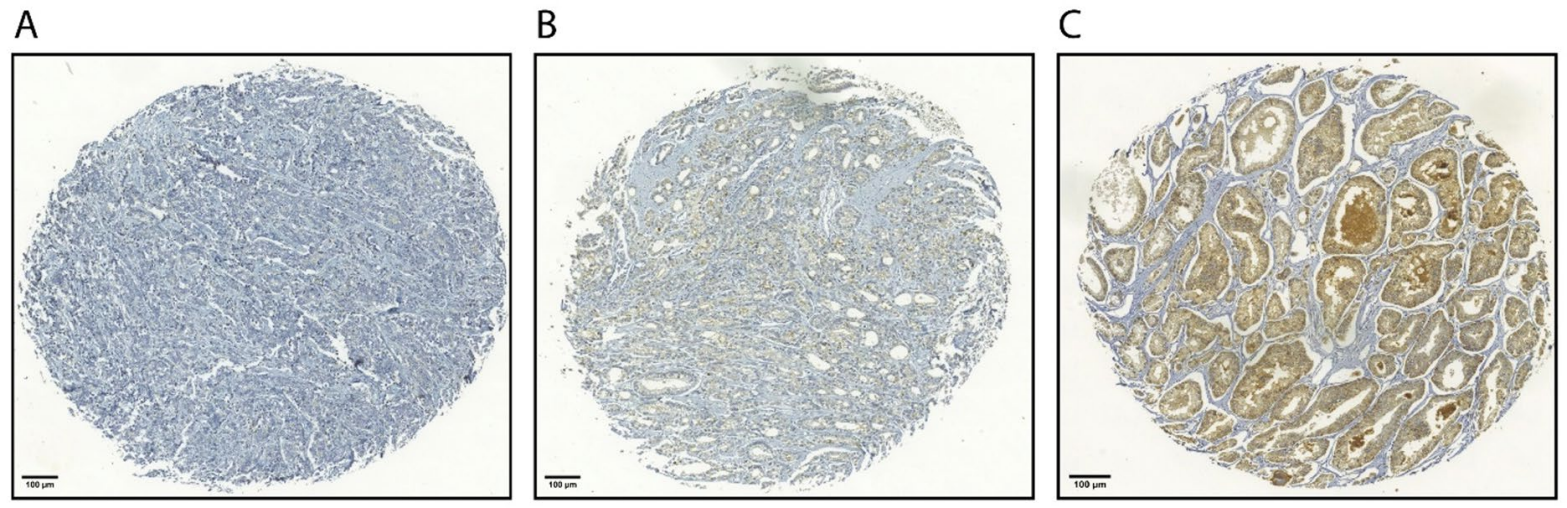
Exemplary GP88 Staining of TMA spots of high-risk PCa. (**A**): Weak GP88 staining with H-Score = 4.42 (1st quartile); (**B**): Strong GP88 staining with H-Score = 111.06 (3rd quartile), (**C**): Very strong GP88 staining with H-Score = 225.28 (4th quartile). Scale bars represent 100 μm.

A cohort of 94 PCa patients what is part of the historical EMPaCT cohort was evaluated for their GP88 protein expression by immunohistochemistry (IHC). GP88 protein expression was detected in the cytoplasm and assessed with an H score, as described in the Material and Methods section. Figure 1 provides representative photomicrographs of GP88 stained biospecimens. The H-score had a range from 3.69 to 252.51 with a mean of 117.77 and a median of 109.28. When separating the H-score in quartiles, we defined a weak GP88 staining for a H-score ≤ 74.8, a moderate GP88 staining for a H-score > 74.8 and ≤ 106.3, a strong GP88 staining for H-score > 106.3 and ≤ 158.5 and a very strong GP88 staining for a H-score > 158.5 (Fig. 1).

**Table 1:** Clinico-pathological data of the PCa patients.

	*N*
All PCa patients	94
Age at diagnosis, years; median (range)	67 (43 − 78)
Pathological tumor stage (pT)	
pT2	12
pT3	66
pT4	16
Pathological node stage (pN)	
pN0	57
pN1	37
Follow up time, months; median (range)	82.5 (1-146)
Overall survival (OS)	
Alive	84
Deceased	10
Disease specific survival	
Yes	87
No	3
Unknown	4
PSA progress	
Yes	29
No	65
Clinical progress	
Yes	13
No	81
Preoperative PSA, ng/ml; median (range) (median (range)	36.9 (20–153)
Gleason score (GS) prostatectomy specimen	
GS 5	18
GS 6	22
GS 7	20
GS 8	14
GS 9	9
Unknown	11
Surgical margin status	
R0	30
R1	48
Unknown	16
Adjuvant hormone deprivation	
Yes	75
No	19
Adjuvant radiation	
Yes	5
No	89

First, we tested whether GP88 staining was associated with clinico-pathological parameters by correlation tests (Spearman’s bivariate correlation test). We tested the clinico-pathological parameters: age at surgery, pathological tumor stage (pT), pathological lymph node stage (pN), preoperative serum PSA, Gleason score of the prostatectomy specimen, surgical margin status, Adjuvant androgen deprivation therapy within 3 months after surgery, Adjuvant radiation therapy of the prostatic bed within 3 months after surgery. There was no correlation of the GP88 H-score with the clinico-pathological parameters after Bonferroni correction.

### GP88 expression and association with survival

GP88 staining was not associated with OS, DFS or RFS. Next, we studied the association with the clinical progression-free survival (CPFS), covering the interval to disease recurrence or metastasis (Table 2). The CPFS was analyzed in an ROC analysis. The GP88 H-score allowed modestly to distinguish between clinical progression and no clinical progression with an area under the curve (AUC) of 0.636 (sensitivity of 53.8% and a specificity of 77.8%), when stratified according to the data-driven optimal cutoff at GP88 H-score < 157 vs. GP88 H-score ≥ 157). Kaplan Meier analysis showed a significant shorter CPFS in the group with the higher GP88 staining (*P* = 0.043; Suppl. Fig. 1). When separating the GP88 staining in two age groups by the median (≤ 67 years vs. > 67 years), the higher GP88 staining was associated with a shorter CPFS only in the elder group (> 67 years; *P* = 0.020) but not in the younger age group. After separating the GP88 staining in the tumor stage groups (pT1, pT3 and pT4) the higher GP88 staining was associated with a shorter CPFS only in the pT3 group (*P* = 0.008) but not in the other pT groups. When separating the GP88 staining in Gleason score of the prostatectomy specimens, a higher GP88 staining was associated with a shorter CPFS in the GS5 and the GS6 groups (*P* = 0.033 and *P* = 0.030) but not in the other GS groups. However, since in the GS5 and the GS6 groups no clinical progress occurred a further confirmation in a larger patient cohort is suggested.

**Table 2:** Kaplan-Meier analysis: Association of GP88 staining with mean CPFS in all patients and in defined patient subgroups.

Kaplan-Meier analysis			
GP88	*N*	CPFS	
H-score < = 157vs > 157			
		Months	p-value
All patients	94	113.96 vs. 132.32	0.043
Patient groups			
Age > 67years	43	92.83 vs. 139.92	0.020
Tumor stage pT3	66	113.17 vs. 138.55	0.008
GS5	18	n.d. ^1^	0.033
GS6	22	n.d. ^1^	0.030
No Adjuvant radiation	89	112.06 vs. 135.69	0.009

^1^n.d. since in the H-score group ≤ 157 no clinical progress occurred.

When separating the GP88 staining according to the adjuvant radiation therapy (yes or no), the higher GP88 staining was associated with a shorter CPFS only in the patient group without radiation therapy (*P* = 0.009), considering that only four patients received an adjuvant radiation therapy. When separating the GP88 staining according to the median preoperative PSA level at diagnosis (level: ≤36.9 vs. >36.9ng/ml), the higher GP88 staining was only as trend associated with a shorter CPFS in the patient group with PSA > 36.9ng/ml (*P* = 0.051) but not for patients with lower preoperative PSA levels. There was no significant association of the GP88 staining with CPFS at separating by the pN, resection margin or adjuvant radiation.

When performing a multivariate Cox’s regression analysis with backward variable selection for the association of the clinical factors with the CPFS, only the preoperative PSA level was significantly associated (RR = 5.287 95CI:1.172–23.86; *p* = 0.030). However, none of the other clinical factors (age at operation, pT, pN, GS of the specimen, resection margins, adjuvant hormone deprivation, adjuvant radiation) was significantly associated with CPFS.

A multivariate Cox’s regression analysis adjusted for preoperative PSA level showed that higher GP88 staining was significantly and independently associated with a shorter CPFS (RR = 2.98, 95%CI: 1.00-8.89; *P* = 0.049; Table 3).

### Correlation of GP88 protein expression and presence of immune cells

On the TMA, in addition to the high Gleason score regions, corresponding low Gleason score regions could be analyzed for their GP88 staining in 58 cases. GP88 staining did not significantly differ between high and low Gleason regions. But cases with an H-score ≤ 157 in the low Gleason region showed a higher presence of immune cells than cases with an H-score > 157 (*P* = 0.008; Fisher’s exact test; Suppl. Tables 1 and Suppl. Fig. 2), what may suggest a somewhat immune cell suppressive effect of GP88.

**Table 3:** Univariate and Multivariate Cox’s regression analyses: Association of GP88 staining and preoperative PSA level with CPFS.

	*N*	Univariate Cox’s regression analysis		Multivariate Cox’s regression analysis	
		CPFS		CPFS	
		RR (95%CI)	p-value	RR (95%CI)	p-value
GP88					
H-score ≤ 157vs. >157	92	2.92 (0.98–8.69)	0.054	2.98 (1.00-8.89)	**0.049**
Preoperative PSA level					
(median: ≤36.9 vs. >36.9ng/ml	92	5.29 (1.72–23.86)	**0.030**	5.38 (1.19–24.29)	**0.029**

Significant p-values are marked in bold face.

## Discussion

High-risk PCa, defined according to clinical parameters such as PSA value, Gleason score or Tumor stage pose a substantial clinical challenge. Patients with these tumors are at high risk to progress towards CRPC or mCRPC. Based on the EMPaCT multi-center database, a preoperative risk-stratification of high-risk PCa patients by clinical parameters as preoperative PSA, tumor stage and ISUP grade has been reported^6^.

However, new biomarkers, beyond clinico-pathological parameters, that may better predict prognosis of high-risk PCa are still needed. In this study, we characterized the GP88 protein expression in a cohort of high-risk PCa by immunohistochemistry and associated our findings to prognosis.

GP88 is well known as growth and proliferation factor in different cancers^11^. It can activate and/or regulate the ERK1/2 and the AKT/PI3K pathways in many different, malignant tumors^11^.

We could previously show that elevated serum levels of GP88 are associated with poorer overall survival of PCa patients^20^. In addition, GP88 detected by immunohistochemistry is a prognostic marker for primary PCa. Higher GP88 protein expression was significantly associated with a shorter overall, disease-specific and relapse free survival in PCa^21^.

GP88 is a marker for metastasis especially in breast cancer^11^. However, we could show for the first time that it is also associated with clinical cancer progression (recurrence and metastasis) in high-risk PCa.

Elevated GP88 expression was associated in Kaplan-Meier analysis with a shorter clinical progression free survival (CPFS) in the complete patient cohort. This result is in line with our previous finding for prognosis in PCa patients^20,21^ and adds knowledge concerning metastatic progression in high-risk PCa patients. In addition, we find that in elder PCa patients (> 67 years), a high expression of GP88 above the optimal H-score cutoff of 157 was associated with shorter CPFS (*P* = 0.020). This finding is somewhat in contrast, to our previous finding that associated elevated GP88 with higher risk especially in younger PCa patients. However, this could be reasoned by the fact that GP88 may play a different role at tumorigenesis in younger PCa patients than at tumor progression in elder PCa patients. Since tumor stage is a well-known prognostic parameter in PCa^3,29^, we stratified our high-risk PCa patients according to the tumor stage and found in the pT3 group a significant association between very high GP88 expression and CPFS. This finding supports our previous result that the association of GP88 with prognosis is dependent on the tumor stage^21^. Interestingly, in contrast to the tumor stage, the GP88 protein expression was associated with shorter CPFS also in lower Gleason scores (GS 5 and GS 6) at taking into account the limited number of patients in these Gleason scores. This finding could be of prognostic relevance since GS6 PCa (ISUP grade group 1) are considered as having an excellent prognosis. However, these tumors display an infiltrative growth, loss of the basal cell layer, cell atypia - that fulfills all the pathological-anatomical criteria of a malignant epithelial tumor - an invasive adenocarcinoma^30^. However, in addition to the clinical parameters (cT, PSA level), old and novel biomarkers including molecular markers [reviewed in^3,7,9^] may also help in prognostic stratification of high-risk PCa with lower GS.

We could show that the GP88 protein expression, stratified by the optimized, data-driven cutoff, and the preoperative PSA level were independent prognostic factors, associated with shorter CPFS. Our historic patient cohort was limited and CPFS event rates of only 13 events had an impact on the robustness of the analyses, as demonstrated by relatively large confidence intervals. Nevertheless, our findings support well-known results of several other studies. An early study showed that the pre-diagnostic PSA level was the only independent factor predicting tumor recurrence^31^. A high pre-diagnostic PSA level was regarded as sufficient to include PCa patients with otherwise low risk features into a prognostic group comparable with the classical high-risk PCa patients^32^. In a cohort of localized prostate cancer, including high-risk tumors, undergoing definitive treatment by brachytherapy, also the preoperative PSA level appeared as independent risk factor for progression-free survival^33^.

Interestingly, a lower GP88 staining in corresponding low Gleason lesions was correlated with the presence of immune cells what suggests an immune cell suppressive effect of GP88. Our finding is somewhat in line with results in breast cancer, where GP88 induces immune escape via up-regulating PD-L1 expression on tumor-associated macrophages (TAMs) and by promoting CD8 + T cell exclusion^34^. Well known is that tumor-associated PD-L1 promotes T-cell apoptosis^35^. The relation between GP88 and PD-L1 may open a new possibility to inhibit immune escape via targeting GP88.

An antisense cDNA-mediated knock-down of GP88 was able to reduce cell proliferation and tumor formation in vivo in a syngeneic C3H mice model for a teratoma PC cell line^36^. In addition, an inhibition of cell proliferation by targeting GP88 has been shown in vitro in several tumor entities such as breast, bladder, colorectal, hepatic and cervical cancers [reviewed in^11,37^]. Moreover, treatment of triple negative breast cancer cell lines with the anti-progranulin (GP88) antibody AG01 resulted in inhibition of proliferation and migration in vitro and tumor growth in vivo^38^. A first-in-class, first-in-human phase 1 Study with the Anti-Progranulin/GP88 Antibody AG01 in advanced solid tumor malignancies (NCT05627960) is on-going^39^.

Modulation of GP88 expression may also play a role in future treatment of a neurodegenerative disease called frontotemporal dementia (FTD), what is an early onset form of dementia. Mutations in the GRN (GP88) gene are the genetic cause of FTD^40^. Mutations mostly result in a loss of function of GP88, therefore enhancing progranulin expression may stop disease progression^41^. However, balancing of GP88 expression may be of very high importance. Recently, Kusakari et al. could show that an overexpression of GP88 in a neuroblastoma cell model induced in vitro endoplasmic reticulum stress and apoptotic cell death. They suggest that a continuous increase in GP88 expression through viral vectors or genetic manipulation can be neurotoxic^42^.

Our study has limitations. It is a retrospective study on historic samples of high-risk PCa patients. Therefore, the number of patients available for statistical analysis is also limited and their treatment regimen was subject to change over time. We used a data-driven approach to define an optimized cutoff value for the GP88 staining, which poses a risk of over-fitting. Therefore, our study has to be complemented by larger studies on recent high-risk PCa patients. In addition, although the biomarkers Ki67 and PTEN have been demonstrated to be of prognostic relevance for PCa patients^43,44^, these markers were not studied in our cohort yet. This investigation is planned in future studies.

Altogether, GP88 protein positivity appears as an independent prognostic factor for clinical progression in high-risk PCa patients. Interestingly, a lower GP88 staining in corresponding low Gleason lesions was correlated with the presence of immune cells suggesting an immune cell suppressive effect of GP88.

## Supplementary Information

Below is the link to the electronic supplementary material.

Supplementary Material 1

## Data Availability

The datasets used and/or analyzed during the current study are available from the corresponding author on reasonable request.

## Abbreviations

ADT: Adjuvant androgen deprivation therapy
AR: Androgen receptor
CPFS: Clinical progression free survival
GP88: Progranulin
GS: Gleason score
IHC: Immunohistochemistry
IRS: Immunoreactive score
OS: Overall survival
PCa: Prostate cancer
pN: Pathological lymph node stage
Pre_PSA: Preoperative serum PSA
pT: Pathological tumor stage
RFS: Relapse-free survival
RR: Relative risk
TAMs: Tumor-associated macrophages
TMA: Tissue micro array
