# Supplementary material for: Expression of progranulin (GP88) protein appears as an independent prognostic factor for clinical progression in high-risk prostate cancer patients
Source: Sci Rep. 2026 Jun 11;16:18179. doi: 10.1038/s41598-026-52197-0 (PMC13260916; doi:10.1038/s41598-026-52197-0)
Supplement: Supplementary file 1 — Supplementary Material 1 [file 41598_2026_52197_MOESM1_ESM.docx]

**Supplementary Material**

**Expression of GP88 (progranulin) protein appears as an independent prognostic factor for clinical progression in high risk prostate cancer patients**

**Renata Dubrovska ^1,^**^$^**, Markus Eckstein ^2,3,^**^$^**, Rudolf Jung ^2,3^, Charis Kalogirou ^4^, Burkhard Kneitz ^4^, Martin Spahn ^5^,** : **Marianna** **Kruithof-de Julio ^6^,** **Ginette Serrero ^7,8^ , Binbin Yue ^8^, Carol Geppert ^2,3^, Robert Stöhr ^2,3^, Arndt Hartmann ^2,3^, Bernd Wullich ^1,3^, Verena Lieb ^1,3^, Helge Taubert ^1,3,^**^$^**,** **and Sven Wach ^1,3,^**^$,^*****

**Supplementary Material and Methods**

***Bibliographic information for the validation of the*** ***GP88/PGRN antibody***

The specificity of the GP88/PGRN antibody has been validated by pathologists when staining formalin-fixed paraffin embedded breast cancer tissue sections [1].

Tissue sections from formalin-fixed, paraffin embedded A475 cells negative for progranulin expression do not stain with GP88/PGRN antibodies whereas tissue section from paraffin embedded O4 cells overexpressing progranulin by transfection with Progranulin cDNA stain strongly with the GP88/PGRN antibody [2].

Additionally, cells where progranulin expression had been inhibited by siRNA transfection showed a decrease of GP88 protein level of expression measured using the GP88/PGRN antibody [3].

**Suppl. Table 1:** Contingency table of H-score and immune cells

|  | | **immune cells 0 vs. >0** | | **Sum** |
| --- | --- | --- | --- | --- |
|  |  | 0 | >0 |  |
| **H-score <=157** | number | 5 | 38 | 43 |
|  | percentage | 8.6 | 65.5 | 74.1 |
| **H-score >157** | number | 7 | 8 | 15 |
|  | percentage | 12.1 | 13.8 | 25.9 |
|  |  |  |  |  |
| **Sum** | number | 12 | 46 | 58 |
|  | percentage | 20.7 | 79.3 | 100 |

Cases with an H-score ≤ 157 in the low Gleason region showed a higher presence of immune cells than cases with an H-score > 157 (P=0.008; Fisher’s exact test).

**Suppl. Figure 1**


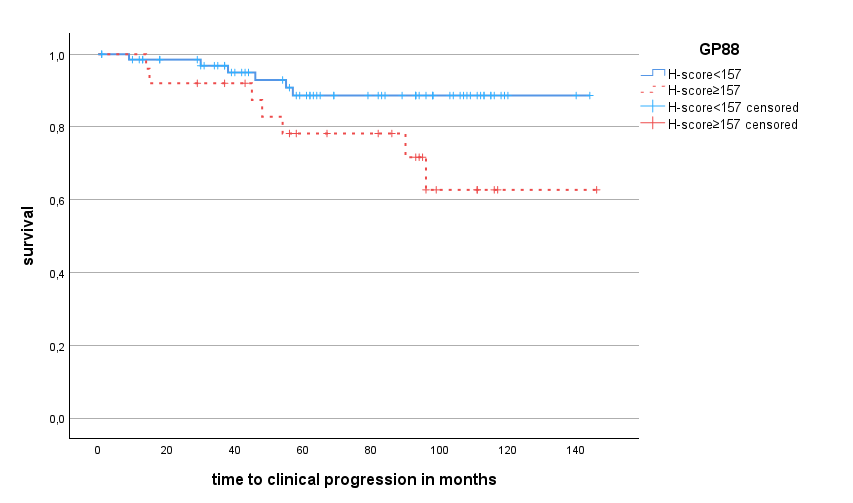


Suppl. Figure 1. Kaplan–Meier analysis: Association of GP88 staining with clinical progression free survival (CPFS) in all PCa patients. GP88 protein expression was significantly associated with CPFS (p = 0.043; log rank test).

**Suppl. Figure 2**


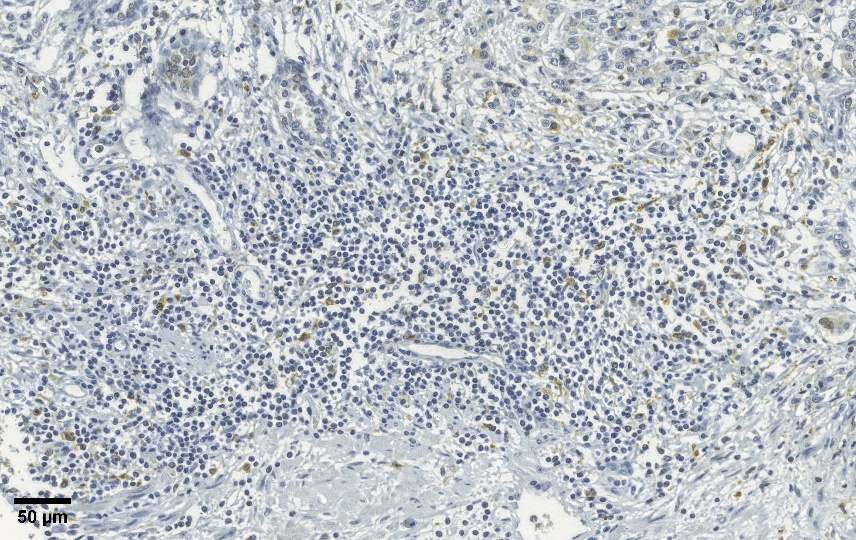


Suppl. Figure 2. Detection of GP88 protein and immune cells. A low expression of GP88 is correlated with the presence of immune cells. Scale bar represents 50µm.

**Additional references**

1. Serrero G and Ioffe O, Expression of the novel autocrine growth factor PC-Cell Derived Growth Factor in human breast cancer tissue. Human Pathology. 2003;34:1148. DOI: 10.1016/s0046-8177(03)00425-8
2. Serrero G, Hicks D. Immunohistochemical Detection of Progranulin (PGRN/GP88/GEP) in Tumor Tissues as a Cancer Prognostic Biomarker. In: Bateman, A., Bennett, H., Cheung, S. (eds) Progranulin. Methods Mol Biol. 2018;1806:107. DOI: 10.1007/978-1-4939-8559-3_8
3. Guha R, Yue B, Dong J, Banerjee A, Serrero G. Anti-progranulin/GP88 antibody AG01 inhibits triple negative breast cancer cell proliferation and migration. Breast Cancer Res Treat. 2021;86(3):637. DOI: 10.1007/s10549-021-06120-y
